# Clinical, imaging features and outcome in internal carotid artery versus middle cerebral artery disease

**DOI:** 10.1371/journal.pone.0225906

**Published:** 2019-12-05

**Authors:** Changqing Zhang, Yilong Wang, Xingquan Zhao, Liping Liu, ChunXue Wang, Zixiao Li, Yuehua Pu, Xinying Zou, Yuesong Pan, Yongjun Wang

**Affiliations:** 1 Department of Neurology, Beijing Tiantan Hospital, Capital Medical University, Beijing, China; 2 China National Clinical Research Center for Neurological Diseases, Beijing, China; 3 Center of Stroke, Beijing Institute for Brain Disorders, Beijing, China; 4 Beijing Key Laboratory of Translational Medicine for Cerebrovascular Disease, Beijing, China; Instituto Mexicano del Seguro Social (IMSS) HGZ 2, MEXICO

## Abstract

**Background:**

Only a very few studies had compared the differences in topographic patterns of cerebral infarcts between middle cerebral artery (MCA) and internal carotid artery (ICA) disease. Besides, the comparison of clinical features and outcomes between MCA and ICA disease had rarely been reported.

**Objectives:**

To compare the clinical, imaging features and outcome of MCA versus ICA disease.

**Methods:**

We prospectively enrolled 1172 patients with noncardiogenic ischemic stroke in ipsilateral ICA or MCA territory. Clinical, neuroradiologic and outcome of the two groups were compared in this observational cohort study.

**Results:**

The ICA group more frequently presented with decreased alertness, gaze palsy, aphasia, and neglect than the MCA group at admission, and more often had higher National Institute of Health stroke scale score at admission and discharge. Meanwhile, the ICA group more frequently had multiple acute infarcts, watershed infarcts, territorial infarct, small cortical infarct, and responsible artery stenosis ≥70%. Whereas penetrating artery infarct and parent artery occluding penetrating artery was more often associated with MCA disease. The ICA group more frequently had inhospital complications of pneumonia and deep vein thrombosis, more often had disability at discharge, and had more recurrent ischemic stroke or transient ischemic attack in 1 Year. Multivariable logistic regression identified male (OR, 1.99; 95% CI, 1.30 to 3.05; P = 0.002), history of coronary heart disease (OR, 1.85; 95% CI, 1.03 to 3.32; P = 0.041), multiple acute infarcts (OR, 4.18; 95% CI, 2.07 to 8.45; P<0.0001), and territorial infarct (OR, 2.23; 95% CI, 1.52 to 3.27; P<0.0001) was more often associated with ICA territory disease.

**Conclusions:**

The clinical, radiologic characteristics and outcome are distinctively different between ICA and MCA disease. Compared to MCA disease, ICA disease has more serious clinical and radiologic manifestation, and poorer outcome.

## Introduction

Several studies have found the differences in the racial distribution of middle cerebral artery (MCA) disease and internal carotid artery (ICA) disease. MCA disease is more common in Asian, Hispanic, and African-American populations, whereas ICA disease is more frequently found in white populations [1–3]. Although the topographic patterns of cerebral infarcts in MCA or ICA disease were individually described in many previous studies [4–6], only a very few studies had compared the differences in topographic patterns of cerebral infarcts between MCA and ICA disease [7]. Besides, the comparison of clinical features and outcomes between MCA and ICA disease had rarely been reported. Hence, in this study, we made a comparative analysis of clinicoradiologic characteristics and outcomes between MCA and ICA disease in China. We think the study will help to understand the differences in clinical, imaging features and outcome between the two diseases, and help to judge the responsible artery and etiology subtype of ischemic stroke according to the clinical and imaging features. Meanwhile, this will help with their diagnosis, treatment and prognosis estimation of MCA disease and ICA disease.

## Methods and subjects

### Ethics statement

This study was approved in writing by the ethics committee of the Beijing Tian Tan Hospital of Capital Medical University and was performed in accordance with the guidelines of the Helsinki Declaration. After ethical approval of Tiantan Hospital was obtained and approved in writing by the other 21 participating hospitals (including Beijing Tongren Hospital of Capital Medical University; Shanghai Jiaotong University Affiliated Sixth People’s Hospital; Shanghai Pudong New Area People’s Hospital; Tianjin Huanhu Hosptial; Shanxi Provincial People’s Hospital; The First Affiliated Hospital of Xiamen University; Xiangya Hospital Central-South University; Chengdu No.3 People’s Hospital; The First Affiliated Hospital of Jinan University; Guangzhou City Peoples First Hospital; Guangdong Hospital of Traditional Chinese Medicine; Handan Central Hospital; Handan First People’s Hospital; Qingdao Municipal Hospital; The First Affiliated Hospital of Zhengzhou University; The First Affiliated Hospital of Zhejiang University; The First Affiliated Hospital of Wenzhou Medical College; Affiliated Kailuan Hospital, North China Coal Medical College; The First Affiliated Hospital of Beifang Medical College; Shijiazhuang Center Hospital; The first affiliated hospital of Hebei North University.), the ethical approval took effect automatically in each center. All patients or their legal representatives provide their written informed consent form. All information was kept confidential. All authors don’t have access to information that could identify individual participants during or after data collection.

### Subjects

Chinese IntraCranial AtheroSclerosis (CICAS) Study is a prospective, multicenter, hospital-based cohort study. Clinical and imaging data were prospectively collected from consecutive patients with ischemic stroke (IS) or transient ischemic attack (TIA) in 22 Chinese general hospitals. From October 2007 to June 2009, 2864 patients with noncardioembolic ischemic cerebrovascular diseases were enrolled in CICAS [3]. Among them, 1172 patients with noncardiogenic IS in ipsilateral ICA or MCA territory enrolled into the current study.

Patients enrolled into the study had the onset of symptoms within 7 days and were between 18 and 80 years old. Patients were excluded if they were clinically unstable, required close monitoring, disabled before admission (modified Rankin scale >2), physically or subjectively unable to comply with magnetic resonance imaging (MRI). We excluded patients with cardioembolic risk factors (atrial fibrillation, valvular heart disease, postcardiac valve replacement, etc) and patients with undetermined causes or other causes. Patients who were diagnosed as TIA, patients without available MR images identifying new cerebral infarct or responsible artery for acute cerebral infarcts, patients with IS involving posterior circulation or bilateral anterior circulation or ipsilateral anterior cerebral artery territory, patients with IS involving both anterior and posterior circulation, patients who received intravenous or intra-arterial thrombolysis, and patients who underwent angioplasty or stent implantation of intracranial or extracranial artery were also excluded. We estimated the sample size needs in the cohort study based on the expected rates of the outcome of interest.

### Clinical information assessment

The clinical information collected included age, sex, hypertension (defined as a history of hypertension or diagnosed at discharge), diabetes mellitus (defined as a history of diabetes mellitus or diagnosed at discharge), hyperlipidemia (defined as low-density lipoprotein cholesterol ≥2.6 mmol/L at the time of admission or a history of hyperlipidemia or received lipid-lowering treatments or diagnosed at discharge), history of IS or intracerebral hemorrhage (ICH), history of coronary heart disease (CHD, defined as a history of myocardial infarction or angina pectoris), National Institutes of Health stroke scale (NIHSS) score at admission and discharge, and Modified Rankin Scale (mRS) before stroke and at discharge. Smoking history, current or previous smokers (continuously smoking ≥1 cigarette a day for 6 months), and history of heavy alcohol use (drinking >2 U/d on average for men or >1 U/d on average for women) were also collected. Uses of antithrombotics within 48 hours of admission, at discharge, and in 1 year after stroke onset were recorded. The frequency of primary symptoms and signs at admission, major complications in hospital were also recorded.

### MRI analysis

All 1172 patients underwent magnetic resonance (MR) imaging on a 3.0 T MR scanner. Imaging sequences obtained included 3-dimensional time-of-flight MR angiography (3D TOFMRA), axial T2-weighted, T1-weighted imaging, fluid-attenuated inversion recovery sequences, and diffusion weighted imaging (DWI). All above sequences except MRA had 5mm slice thickness and 1.5mm interslice gap. MR images were viewed by using software (RadiAnt DICOM Viewer1.0.4.4439, Medixant Ltd, poznan, Poland).

The stenotic degree of intracranial and extracranial vessels were examined using 3D TOF MRA, and contrast enhanced MRA (CEMRA) or duplex color Doppler ultrasound, respectively. Responsible artery for acute cerebral infarct was defined as intracranial or extracranial artery responsible for acute cerebral infarcts according to the distribution characteristic of infarct lesions and examination results of MRA and CEMRA or color Doppler ultrasound [8]. MCA was confirmed as the responsible artery for acute IS when new ischemic lesions confirmed by DWI sequence distributed within ipsilateral MCA territory or watershed areas, and there were no stenosis in ipsilateral carotid artery [9]. ICA was confirmed as the responsible artery for acute IS when new ischemic lesions confirmed by DWI sequence distributed within ipsilateral ICA territory or watershed areas, and there were >50% stenosis in ipsilateral ICA and no stenosis in ipsilateral MCA [7]. But when ipsilateral ICA and MCA were both occluded, ICA was also confirmed as the responsible artery for acute IS.

The degree of intracranial stenosis on MRA was calculated using the published method for the Warfarin–Aspirin Symptomatic Intracranial Disease Study [10]. All measurements were made using Wiha DigiMax Digital Calipers 6’ (Germany) with a resolution of 0.01 to 0.03 mm for 0 to 100 mm. The degree of responsible extracranial artery stenosis was estimated with ultrasonographic examination according to the published diagnostic criteria or calculated according to North American Symptomatic Carotid Endarterectomy Trial (NASCET) criteria by CEMRA [11, 12]. According to the severity of the stenosis, we classified the responsible cerebral vessels into 4 groups: <50% or no stenosis, 50% to 69% stenosis, 70% to 99% stenosis, and occlusion groups.

**Fig 1:**
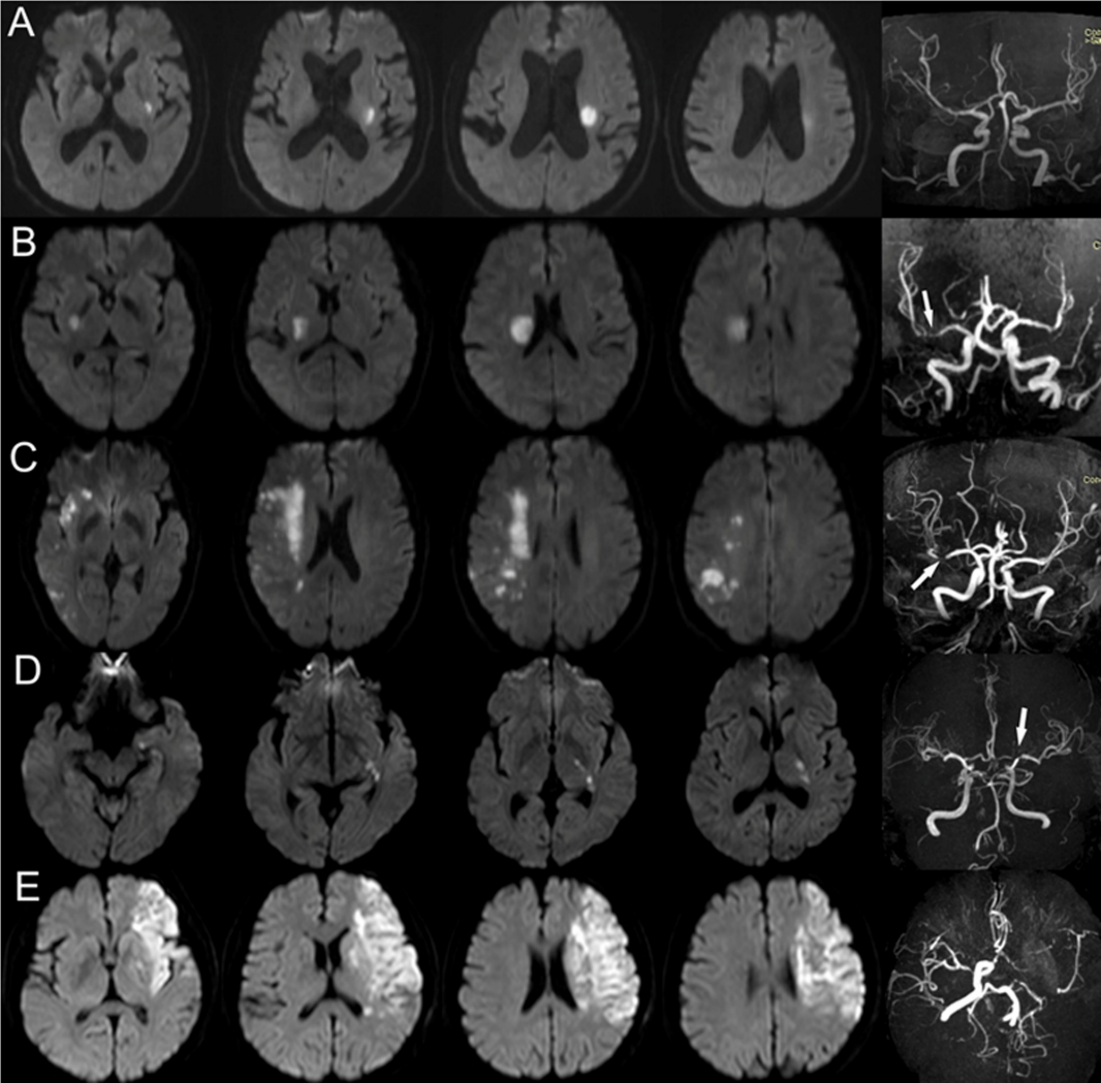
Topographical Distribution of Acute Ischemic Stroke in Internal Carotid Artery (ICA) or Middle Cerebral Artery (MCA) Territory. A. Single acute infarct in left lenticulostriate artery of MCA territory, without obvious stenosis of left MCA. Small artery occlusion was the most possible etiologic subtype. B. A single acute infarct in right lenticulostriate artery territory, with obvious stenosis in M1 segment of RMCA. Large artery atherosclerosis (LAA) and parent artery occluding penetrating artery was the etiologic subtype and most possible stroke mechanism. C. Multiple acute infarcts (including internal watershed infarcts, cortical watershed infarcts, and small cortical infarct) in right MCA territory, with obvious stenosis in M1 segment of right MCA. The etiologic subtype was considered LAA. D. Single acute infarct in left anterior choroidal artery of ICA territory, with obvious stenosis in C7 segment of left ICA. LAA and parent artery occluding penetrating artery was considered as the etiologic subtype and most possible stroke mechanism. E. Multiple acute infarcts (including territorial infarct and penetrating artery infarct) in left ICA territory with occlusion of left ICA. The etiologic subtype was considered LAA.

Topographical distribution of acute infarcts (including single or multiple acute infarcts, watershed infarcts, small cortical infarct, territorial infarct, and penetrating artery infarct) were evaluated (Fig 1). Multiple acute cerebral infarcts was defined as ≥2 separate lesions that were hyperintense on DWI. Watershed infarcts were defined as infarcts occured at a junction of two (or three) artery territories with arterial collateral circulation. Watershed infarcts were classified as internal watershed infarcts (IWS) and cortical watershed infarcts (CWS). IWS was defined as rosary-like pattern of infarcts arranged in a linear fashion parallel to the lateral ventricle and located in the centrum semiovale or corona radiata. CWS are distinguished as anterior cortical watershed infarcts (ACWS) and posterior cortical watershed infarcts (PCWS) using the method described in the previous study [13]. With the exception of watershed, small cortical infarct was defined as cortical infarct with a maximum diameter of <2 cm. Territorial infarct was defined as a large ischemic lesion with a maximum diameter of ≥2 cm involving the cerebral cortical and subcortical structure in one or more major cerebral artery territories [6].

Etiologic subtypes of IS were classified according to the Stop Stroke Study Trial of Org 10172 in Acute Stroke Treatment (SSS-TOAST) classification criteria [14]. Stroke mechanism of large artery atherosclerosis IS was determined as parent artery occluding penetrating artery if isolated acute infarct located in penetrating artery territory and the parent artery had evidence of plaque or any degree of stenosis [15].

Two radiologists blinded to the clinical details read all MRI scans. Consensus was reached by them if they had disagreement on interpretations.

### Follow up and clinical outcome evaluations

At 3, 6, and 12 months after discharge, patients or their relatives were contacted over the telephone by trained research personnel at Beijing Tian Tan hospital, and were asked whether patients had new symptoms or hospitalized again with another stroke. The primary outcome was recurrence of IS or TIA in one year.

Recurrence of IS was defined as a new focal neurological deficit of vascular origin lasting >24 hours and without hemorrhage on computed tomography (CT) or MRI of the brain [16]. A recurrent TIA was defined as a new focal neurological deficit sustained for a duration of <24 hours caused by ischemia in the brain or retina. All recurrence of IS or TIA was verified at the index hospitals based on the presence of new neurological deficits documented in the medical records combining with CT or MRI images. An experienced stroke neurologist reviewed the patients’ medical document to ensure a reliable diagnosis of recurrence of IS. In case of an unclear event that was not hospitalized, the case would be adjudicated by a stroke neurologist and the principle investigator. Any death was verified by examining the hospital medical records or local citizen registry.

### Statistics

The Mann–Whitney *U* test was used for comparison of continuous variables with non-normal distribution. χ^2^ test was used for comparison of categorical variables. The baseline relative factors, clinical and imaging features, and outcome were presented according to the responsible artery of IS in ipsilateral ICA or MCA territory. Multivariable logistic regression analysis was used to identify relative factors associated with the responsible artery of IS in ipsilateral ICA or MCA territory. All parameters that were significant by univariate analysis at *P*<0.05 level or likely to have pathophysiologic influence were included in the multivariable logistic regression analysis. All probability values were 2-tailed; *P*<0.05 was considered to be statistically significant. All analyses were performed by using SAS Version 9.1 (SAS Institute, Cary, NC).

## Results

### General patient characteristics

**Fig 2:**
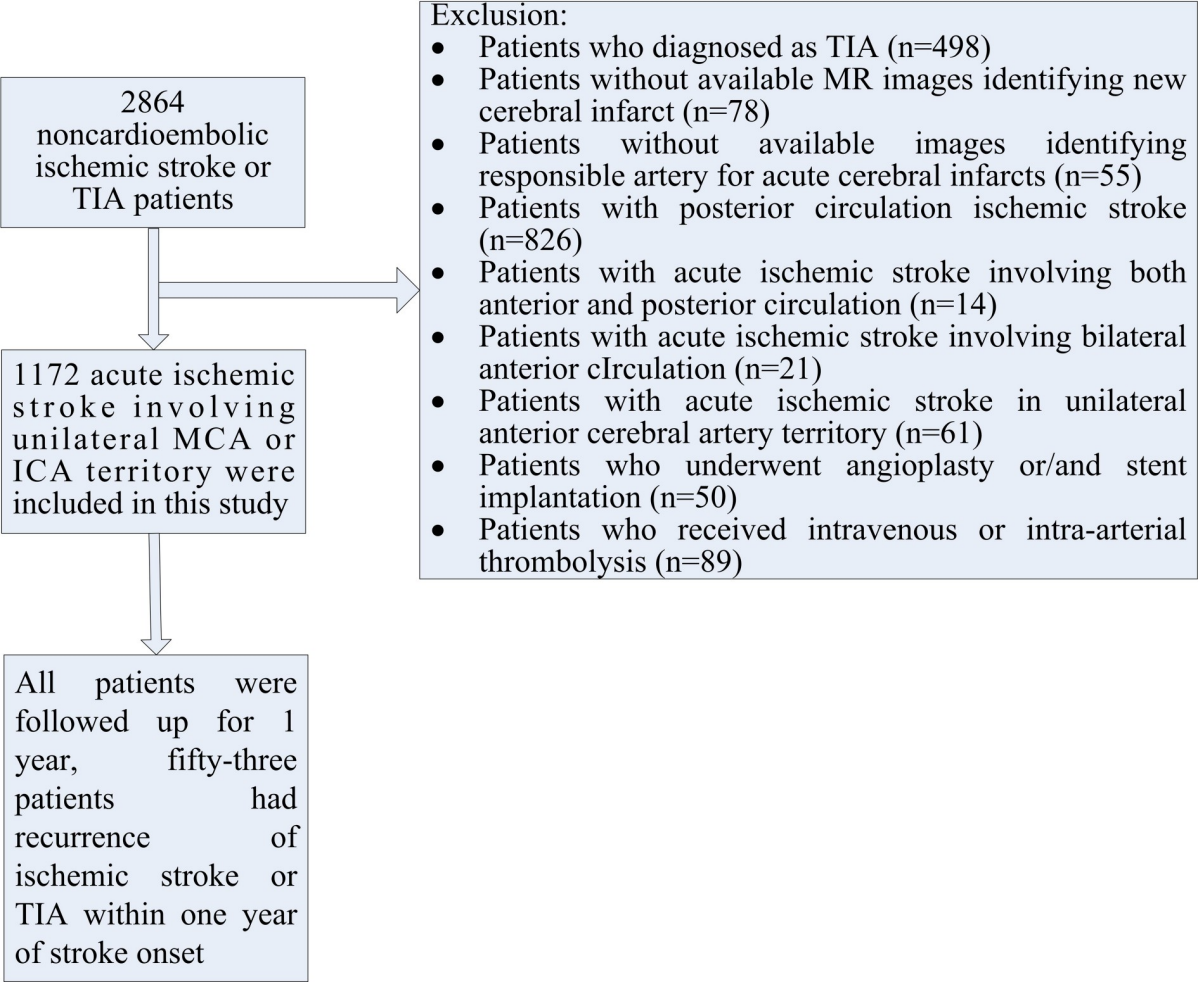
Flow chart of patients enrollment.

The final analysis included 1172 patients with noncardiogenic IS in ipsilateral ICA or MCA territory (Fig 2). Demographic Features of 1172 Patients were presented in Table 1. There were no missing data for each variable of interest. The studied population consisted of 1172 patients (823 men, 349 women) with a mean age of 61.4±11.4 years (range, 19 to 80 years), 246 patients (21.0%) had infarcts in ICA territory, and 926 patients (79.0%) had infarcts in MCA territory. A total of 231 patients underwent CEMRA and 1006 patients underwent ultrasonographic examination, and 65 patients underwent both CEMRA and ultrasonographic examination to assess extracranial carotid disease. As for vascular risk factors, 912 patients (77.8%) had hypertension, 381 (32.5%) had diabetes, 892 (76.1%) had hyperlipidemia. In addition, 297 patients (25.4%) had a history of IS, 79 (6.7%) had a history of coronary heart disease, and 63 patients (5.4%) had repeated TIAs in 3 months before the stroke onset. Admission symptoms and signs of 1172 patients were presented in Table 2. Facial palsy, unilateral limb weakness, dysarthria, and aphasia were the primary symptoms or signs in 841 (71.8%), 704 (60.1%), 534 (45.6%), and 347 (29.6%) patients. The distribution features of infarcts were presented in Table 3. A total of 769 IS patients (65.6%) were due to large artery atherosclerosis (LAA), while 403 (34.4%) were due to small artery occlusion (SAO). Responsible artery stenosis ≥70%, multiple acute infarcts, and watershed infarcts were present in 657 (56.1%), 583 (49.7%), and 533 (45.5%) patients, respectively. All patients were followed up for 1 year, fifty-three patients (4.5%) had recurrence of ischemic stroke or TIA within one year of stroke onset.

**Table 1:** Demographic Features of 1172 patients with acute ischemic stroke in ICA versus MCA territory.

Variables	Total (n = 1172)	ICA(n = 246)	MCA(n = 926)	P value
**Demographics**				
Age, median (IQR), y	62[53,71]	63[55,70]	61[52,71]	0.172
Age ≥65 years	504(43.0)	107(43.5)	397(42.9)	0.861
Male	823(70.2)	196(79.7)	627(67.7)	<0.0001
**Vascular Risk factors**				
Smoking	488(41.6)	120(48.8)	368(39.7)	0.011
Heavy drinking	62(5.3)	17(6.9)	45(4.9)	0.201
Hypertension	912(77.8)	189(76.8)	723(78.1)	0.675
Diabetes mellitus	381(32.5)	83(33.7)	298(32.2)	0.643
Hyperlipidemia	892(76.1)	197(80.1)	695(75.1)	0.100
CHD	79(6.7)	22(8.9)	57(6.2)	0.121
History of ischemic stroke	297(25.4)	79(32.1)	218(23.6)	0.006
History of stroke	314(26.8)	82(33.3)	232(25.1)	0.009
Days from onset to admission	2[1,3]	2[1,4]	2[1,3]	0.263
Days from onset to MR scan	4[2,6]	4[2,7]	4[2,6]	0.342

CHD, coronary heart disease; ICA, internal carotid artery; MCA, middle cerebral artery; IQR, interquartile range; Data are n (%) unless otherwise indicate.

**Table 2:** Admission symptoms and signs of 1172 patients with acute ischemic stroke in ICA versus MCA territory.

Symptoms and signs	Total (n = 1172)	ICA(n = 246)	MCA(n = 926)	P value
Decreased alertness	72(6.1)	33(13.4)	39(4.2)	<0.0001
Gaze palsy	77(6.6)	31(12.6)	46(5.0)	<0.0001
Visual field loss	39(3.3)	10(4.1)	29(3.1)	0.468
Facial Palsy	841(71.8)	188(76.4)	653(70.5)	0.067
Unilateral limb weakness	704(60.1)	157(63.8)	547(59.1)	0.176
Limb Ataxia	100(8.5)	25(10.2)	75(8.1)	0.303
Sensory loss	374(31.9)	91(37.0)	283(30.6)	0.054
Aphasia	347(29.6)	93(37.8)	254(27.4)	0.002
Dysarthria	534(45.6)	98(39.8)	436(47.1)	0.042
Neglect	27(2.3)	13(5.3)	14(1.5)	<0.0001
Dizziness	70(6.0)	17(6.9)	53(5.7)	0.485
Diplopia	15(1.3)	5(2.0)	10(1.1)	0.333
Dysphagia	81(6.9)	19(7.7)	62(6.7)	0.572
Headache	56(4.8)	13(5.3)	43(4.6)	0.675

**Table 3:** Clinical, imaging features and outcome of 1172 patients with acute ischemic stroke in ICA versus MCA territory.

Variables	Total (n = 1172)	ICA(n = 246)	MCA(n = 926)	P value
**Clinical and Imaging Features**				
Admission NIHSS, median (IQR)	4[2,8]	6[3,11]	4[2,8]	<0.0001
Prestroke mRS,median (IQR)	0[0,0]	0[0,0]	0[0,0]	0.139
Prestroke mRS ≧2	33(2.8)	6(2.4)	27(2.9)	0.679
Repeated TIAs before the stroke	63(5.4)	13(5.3)	50(5.4)	0.943
Time from onset to MR scan, median (IQR)	4[2,6]	4[2,7]	4[2,6]	0.105
Multiple acute infarcts	583(49.7)	211(85.8)	372(40.2)	<0.0001
Watershed infarcts	533(45.5)	192(78.0)	341(36.8)	<0.0001
Internal watershed infarcts	477(40.7)	174(70.7)	303(32.7)	<0.0001
Anterior cortical watershed infarcts	288(24.6)	111(45.1)	177(19.1)	<0.0001
Posterior cortical watershed infarcts	309(26.4)	104(42.3)	205(22.1)	<0.0001
Territorial infarct	276(23.5)	125(50.8)	151(16.3)	<0.0001
Small cortical infarct	444(37.9)	172(69.9)	272(29.4)	<0.0001
Penetrating artery infarct	836(71.3)	128(52.0)	708(76.5)	<0.0001
Responsible artery stenosis ≥70%	657(56.1)	211(85.8)	446(48.2)	<0.0001
LAA subtype*	769(65.6)	233(94.7)	536(57.9)	<0.0001
Parent artery occluding penetrating artery	174(23.0)	10(4.3)	164(30.6)	<0.0001
**Performance measures**				
Early Antithrombotics after admission	1136(97.9)	239(98.4)	897(97.8)	0.602
Antithrombotics at discharge	1077(92.4)	220(90.2)	857(93.0)	0.145
Antithrombotics in 1 year	755(66.1)	155(65.4)	600(66.2)	0.811
Statins in 1 year	393(34.4)	80(33.8)	313(34.5)	0.819
**Complications**				
Pneumonia	76(6.5)	25(10.2)	51(5.5)	0.008
Deep vein thrombosis	8(0.7)	8(3.3)	0(0)	<0.0001
Gastrointestinal bleeding	12(1.0)	5(2.0)	7(0.8)	0.078
**Outcome**				
Discharge NIHSS, median (IQR)	2[1,6]	4[1,8]	2[1,5]	<0.0001
Discharge mRS, median (IQR)	2[1,3]	3[1,4]	1[1,3]	<0.0001
Discharge mRS ≧2	606(51.7)	161(65.4)	445(48.1)	<0.0001
Recurrent Ischemic stroke or TIA in 1 Year	53(4.5)	18(7.3)	35(3.8)	0.018
Death in 1 year	29(2.5)	9(3.7)	20(2.2)	0.179

CHD, coronary heart disease; ICA, internal carotid artery; LAA, large artery atherosclerosis; MCA, middle cerebral artery; mRS, the Modified Rankin Scale; NIHSS, National Institutes of Health stroke scale; TIA, transient ischemic attack; IQR, interquartile range; Data are n (%) unless otherwise indicate.

*It is contrary to small artery occlusion subtype of ischemic stroke according to Stop Stroke Study Trial of Org 10172 in Acute Stroke Treatment (SSS-TOAST) classification criteria.

### Comparison of clinical, imaging features and outcome in ICA versus MCA disease

Univariate analysis found that patients with infarcts in ICA territory more often were male, had smoking and a history of IS or stroke. Besides, the ICA group more frequently presented with decreased alertness, gaze palsy, aphasia, and neglect than the MCA group at admission, and more often had higher NIHSS scores at admission and discharge. Meanwhile, the ICA group more frequently had multiple acute infarcts, watershed infarcts, territorial infarct, small cortical infarct, LAA subtype of IS, and responsible artery stenosis ≥70%. Whereas penetrating artery infarct, SAO subtype of IS, and parent artery occluding penetrating artery more often occurred in MCA territory disease. The ICA group more frequently had inhospital complications of pneumonia and deep vein thrombosis, more often had disability at discharge, and had more recurrent IS or TIA in 1 Year.

When adjusted for age, sex, and vascular risk factors, multivariable logistic regression identified male (OR, 1.99; 95% CI, 1.30 to 3.05; P = 0.002), history of CHD (OR, 1.85; 95% CI, 1.03 to 3.32; P = 0.041), multiple acute infarcts (OR, 4.18; 95% CI, 2.07 to 8.45; P<0.0001), and territorial infarct (OR, 2.23; 95% CI, 1.52 to 3.27; P<0.0001) was more often associated with ICA territory disease (Table 4).

**Table 4:** Multivariable logistic regression for relative factors associated with acute ischemic stroke in ICA rather than MCA territory.

Variables	OR (95% CI)	adjusted OR† (95% CI)	P value
Age ≥65 years (yes)	1.026(0.772–1.362)	1.144(0.811–1.613)	0.444
Male (yes)	1.869(1.331–2.626)	1.993(1.301–3.053)	0.002
Smoking (yes)	1.444(1.089–1.916)	1.060(0.731–1.537)	0.760
Drinking (yes)	1.453(0.817–2.587)	1.348(0.671–2.709)	0.401
Hypertension (yes)	0.931(0.666–1.301)	1.104(0.747–1.629)	0.620
Diabetes mellitus (yes)	1.073(0.796–1.446)	1.133(0.801–1.604)	0.480
Hyperlipidemia (yes)	1.336(0.945–1.890)	1.452(0.989–2.133)	0.057
CHD (yes)	1.497(0.896–2.502)	1.846(1.025–3.324)	0.041
History of ischemic stroke (yes)	1.534(1.128–2.087)	1.224(0.859–1.744)	0.264
Admission NIHSS >3 (yes)	1.662(1.232–2.241)	0.819(0.522–1.285)	0.385
Discharge NIHSS >3 (yes)	1.971(1.520–2.557)	1.357(0.858–2.145)	0.192
Discharge mRS ≧2 (yes)	2.166(1.628–2.883)	1.324(0.834–2.103)	0.234
Multiple acute infarcts (yes)	8.978(6.133–13.14)	4.184(2.071–8.452)	<0.0001
Territorial infarct (yes)	5.302(3.909–7.191)	2.226(1.517–3.266)	<0.0001
Small cortical infarct (yes)	5.589(4.111–7.597)	1.160(0.739–1.820)	0.520
Watershed infarcts (yes)	6.100(4.385–8.485)	1.245(0.555–2.796)	0.595
Internal watershed infarcts (yes)	4.969(3.655–6.755)	0.995(0.532–1.861)	0.988
Penetrating artery infarct (yes)	0.334(0.249–0.448)	0.841(0.578–1.224)	0.366

CHD, coronary heart disease; ICA, internal carotid artery; MCA, middle cerebral artery; mRS, the Modified Rankin Scale; NIHSS, National Institutes of Health stroke scale; OR, odds ratio

^†^Multivariable logistic regression adjusted for age, sex, vascular risk factors, clinical and imaging features.

## Discussion

In this study, we found that ICA disease group has more serious clinical and radiologic manifestation, and poorer outcome compared to MCA disease group. MCA is the most common site of intracranial artery stenosis in China [3, 17], so IS in MCA territory is also the most common type of IS caused by intracranial artery disease in China. Our study found that IS in MCA territory is far more common than that in ICA territory. The most common infarction pattern of IS in MCA territory is single infarct, and the most common type of etiology of single infarct is small artery occlusion, followed by large artery atherosclerosis (the underlying mechanism is the parent artery plaque or thrombus occluding penetrating artery). Previous study found that patients with vulnerable symptomatic plaque in MCA more commonly demonstrated an artery-to-artery embolic infarction pattern than the patients with stable symptomatic plaque [17]. Single infarct is the most common infarction pattern of MCA group in our study, so MCA group mostly have no plaque or maybe they only have stable plaque in MCA. However, the most common infarction pattern of IS in ICA group was multiple infarcts, internal watershed infarcts and small cortical infarct, 85.8% IS patients had ≥70% stenosis of responsible artery, large artery atherosclerosis was the main etiological type in 94.7% ICA group patients in our study. Researches found that internal watershed infarcts were mainly caused by hemodynamic compromise, whereas cortical watershed infarcts and small cortical infarcts were mainly caused by embolic pathogenesis[4, 18–20], so the most common underlying stroke mechanisms in ICA group were hemodynamic compromise or artery-to-artery embolism in our study. Research also found that atherosclerotic plaque ulceration in ICA was associated with nonlacunar ischemic stroke [21]. In our study, 211 (85.8%) IS in ICA territory had multiple acute infarcts, and 172(69.9%) IS in ICA territory had small cortical infarct, so most IS in ICA territory were nonlacunar subtype. Therefore, the symptomatic plaques in ICA group maybe are mostly unstable or ulcer plaques in our study.

The proportion of ≧70% degree stenosis of ICA in ICA group was 85.8%, which was much higher than the proportion of ≧70% degree stenosis of MCA in MCA group (48.2%). This can explain why the proportion of small artery occlusion etiological type of IS in MCA group is much higher than that in ICA group. Small artery occlusion etiological type of IS in MCA territory belongs to the category of cerebral small vessel disease. Hypertension is the most important risk factor for cerebral small vessel disease. The proportion of ischemic cerebrovascular disease caused by intracranial atherosclerosis in China is much higher than that in the western white people [1–3], the higher prevalence and lower control rate of hypertension in Chinese may be the main reasons for the above differences [22, 23]. In addition, genetic differences among different races are also possible reasons [24].

The infarct area in ICA group is larger than that in MCA group, and the proportion of territorial infarct is higher. This may be because the volume of ICA plaque is larger than that of MCA plaque, then the volume of exfoliated plaque or thrombus is larger, and therefore the diameter of distal blocked vessel is larger. Meanwhile, the proportion of watershed infarct in ICA group is higher. This may be because the proportion of severe stenosis of ICA in ICA group is higher than the proportion of severe stenosis of MCA in MCA group, and the compensation of cerebral blood flow and collateral circulation in ICA group are worse than those in MCA group. Therefore, the proportion of patients with watershed infarct and internal watershed infarct in ICA group is much higher than that in MCA group.

The area of cerebral infarct in ICA group is relatively larger, and therefore the degree of neurological deficit and the proportion of disability at admission and discharge are also significantly higher in ICA group. Correspondingly, the incidence of pneumonia and deep venous thrombosis in ICA group during hospitalization are also higher than those in MCA group. ICA group have higher proportion of severe stenosis and unstable plaque of responsible artery, cerebral blood flow compensation in ICA group is relatively poorer, and therefore the recurrence of ischemic stroke or TIA within one year after onset is also higher.

### Strengths and limitations

The strengths of our study include the large sample size of patients with noncardiogenic ischemic stroke in ipsilateral ICA or MCA territory, and our use of standardized methods to evaluate the patients’ multiple imaging features such as SSS-TOAST stroke etiologic subtype, degree of responsible artery stenosis, and distribution patterns of cerebral infarcts. Research results from the large, prospective, multicenter, cohort study are helpful to understand the differences between ICA and MCA disease, and to improve the diagnosis and treatment of these patients. There are some limitations in our study. First, patients who were clinically unstable, required close monitoring, disabled before admission, unable to comply with MRI were excluded, these may possibly result in selection bias. Second, some extracranial artery stenosis were calculated by CEMRA, while others were estimated with ultrasonographic examination, these may cause some differences when compared. Third, the ICA sample in our study was heterogenous, because they included extracranial ICA occlusion and intracranial ICA occlusion. The vast majority of ICA occlusion was extracranial, and the majority of pathogenesis of extracranial ICA occlusion was in situ atherosclerotic disease. However, the majority of pathogenesis of intracranial ICA occlusion was embolic. This is a limitation of the study because they represent almost different diseases.

## Conclusions

The clinical, radiologic characteristics and outcome are distinctively different between ICA and MCA territory disease. Compared to MCA disease group, ICA disease group has more serious clinical and radiologic manifestation, and poorer outcome. Meanwhile, the distribution of etiology subtypes between MCA disease and ICA disease was significantly different. We think the study will help to judge the responsible artery and etiology subtype of ischemic stroke according to the different clinical and imaging features of MCA disease and ICA disease. Moreover, this will help with their diagnosis, treatment and prognosis estimation of the two diseases.

## Supporting information

S1 File Data file. (SAV)

S2 File STROBE_checklist. (DOCX)
