# Supplementary material for: Clinical, imaging features and outcome in internal carotid artery versus middle cerebral artery disease
Source: PLoS One. 2019 Dec 5;14(12):e0225906. doi: 10.1371/journal.pone.0225906 (PMC6894760; doi:10.1371/journal.pone.0225906)
Supplement: S2 File — (DOCX) [file pone.0225906.s002.docx]

STROBE Statement—checklist of items that should be included in reports of observational studies

|  | | | Item No. | Recommendation | | Page  No. | | | Relevant text from manuscript |
| --- | --- | --- | --- | --- | --- | --- | --- | --- | --- |
| **Title and abstract** | | | 1 | (*a*) Indicate the study’s design with a commonly used term in the title or the abstract | | 2 | | | We prospectively enrolled, in this observational cohort study |
|  |  |  |  | (*b*) Provide in the abstract an informative and balanced summary of what was done and what was found | | 2 | | | The ICA group more frequently presented with, Multivariable logistic regression identified |
| Introduction | | | | | | | | |  |
| Background/rationale | | | 2 | Explain the scientific background and rationale for the investigation being reported | | 3 | | | Only a very few studies had compared the differences in topographic patterns of cerebral infarcts between atherosclerotic MCA and ICA disease. Besides, the comparison of clinical features and outcomes between atherosclerotic MCA and ICA disease had rarely been reported |
| Objectives | | | 3 | State specific objectives, including any prespecified hypotheses | | 3 | | | we made a comparative analysis of clinicoradiologic characteristics and outcomes between atherosclerotic MCA and ICA disease in China. |
| Methods | | | | | | | | |  |
| Study design | | | 4 | Present key elements of study design early in the paper | | 5 | | | Chinese IntraCranial AtheroSclerosis (CICAS) Study is a prospective, multicenter, hospital-based cohort study. |
| Setting | | | 5 | Describe the setting, locations, and relevant dates, including periods of recruitment, exposure, follow-up, and data collection | | 5,10 | | | Clinical and imaging data were prospectively collected from consecutive patients with ischemic stroke (IS) or transient ischemic attack (TIA) in 22 Chinese general hospitals. From October 2007 to June 2009, 2864 patients with noncardioembolic ischemic cerebrovascular diseases were enrolled in CICAS. At 3, 6, and 12 months after discharge, patients or their relatives were contacted over the telephone. |
| Participants | | | 6 | (*a*) *Cohort study*—Give the eligibility criteria, and the sources and methods of selection of participants. Describe methods of follow-up  *Case-control study*—Give the eligibility criteria, and the sources and methods of case ascertainment and control selection. Give the rationale for the choice of cases and controls  *Cross-sectional study*—Give the eligibility criteria, and the sources and methods of selection of participants | | 5,10 | | | Patients enrolled into the study had the onset of symptoms within 7 days and were between 18 and 80 years old. Patients were excluded if they were; At 3, 6, and 12 months after discharge, patients or their relatives were contacted over the telephone. |
|  |  |  |  | (*b*) *Cohort study*—For matched studies, give matching criteria and number of exposed and unexposed  *Case-control study*—For matched studies, give matching criteria and the number of controls per case | |  | | |  |
| Variables | | | 7 | Clearly define all outcomes, exposures, predictors, potential confounders, and effect modifiers. Give diagnostic criteria, if applicable | | 6,7,8,10 | | | The clinical information collected included, MCA was confirmed as the responsible artery for acute IS when, ICA was confirmed as the responsible artery for acute IS when, The degree of intracranial stenosis on MRA was calculated using, The primary outcome was recurrence of IS or TIA in one year. |
| Data sources/ measurement | | | 8* | For each variable of interest, give sources of data and details of methods of assessment (measurement). Describe comparability of assessment methods if there is more than one group | | 7,8,10 | | | Etiologic subtypes of IS were classified according to; Multiple acute cerebral infarcts was defined as, Watershed infarcts were defined as, small cortical infarct was defined as. |
| Bias | | | 9 | Describe any efforts to address potential sources of bias | | 10 | | | Two radiologists blinded to the clinical details read all MRI scans. Consensus was reached by them if they had disagreement on interpretations. |
| Study size | | | 10 | Explain how the study size was arrived at | | 6 | | | We estimated the sample size needs in the cohort study based on the expected rates of the outcome of interest. |
| Quantitative variables | | 11 | | | Explain how quantitative variables were handled in the analyses. If applicable, describe which groupings were chosen and why | | 11 | | The Mann–Whitney *U* test was used for comparison of continuous variables with non-normal distribution. |
| Statistical methods | | 12 | | | (*a*) Describe all statistical methods, including those used to control for confounding | | 11 | | χ^2^ test was used for comparison of categorical variables; Multivariable logistic regression analysis was used to |
|  |  |  |  |  | (*b*) Describe any methods used to examine subgroups and interactions | | 11 | | The baseline relative factors, clinical and imaging features, and outcome were presented according to the responsible artery of IS in unilateral ICA or MCA territory. Multivariable logistic regression analysis was used to |
|  |  |  |  |  | (*c*) Explain how missing data were addressed | | 12 | | All patients were followed up for 1 year |
|  |  |  |  |  | (*d*) *Cohort study*—If applicable, explain how loss to follow-up was addressed  *Case-control study*—If applicable, explain how matching of cases and controls was addressed  *Cross-sectional study*—If applicable, describe analytical methods taking account of sampling strategy | | 12 | | All patients were followed up for 1 year, |
|  |  |  |  |  | (*e*) Describe any sensitivity analyses | | N/A | |  |
| Results | | | | | | | | | |
| Participants | | 13* | | | (a) Report numbers of individuals at each stage of study—eg numbers potentially eligible, examined for eligibility, confirmed eligible, included in the study, completing follow-up, and analysed | | 11 | | The final analysis included 1172 patients with noncardiogenic IS in unilateral ICA or MCA territory (Figure 2). |
|  |  |  |  |  | (b) Give reasons for non-participation at each stage | | 11 | | Fig 2 |
|  |  |  |  |  | (c) Consider use of a flow diagram | | 11 | | Fig 2 |
| Descriptive data | | 14* | | | (a) Give characteristics of study participants (eg demographic, clinical, social) and information on exposures and potential confounders | | 11 | | Demographic Features of 1172 Patients were presented |
|  |  |  |  |  | (b) Indicate number of participants with missing data for each variable of interest | | 11 | | There were no missing data for each variable of interest |
|  |  |  |  |  | (c) *Cohort study*—Summarise follow-up time (eg, average and total amount) | | 12 | | All patients were followed up for 1 year |
| Outcome data | | 15* | | | *Cohort study*—Report numbers of outcome events or summary measures over time | | 12 | | Fifty-three patients had recurrence of ischemic stroke or TIA within one year of stroke onset |
|  |  |  |  |  | *Case-control study—*Report numbers in each exposure category, or summary measures of exposure | | N/A | |  |
|  |  |  |  |  | *Cross-sectional study—*Report numbers of outcome events or summary measures | | N/A | |  |
| Main results | | 16 | | | (*a*) Give unadjusted estimates and, if applicable, confounder-adjusted estimates and their precision (eg, 95% confidence interval). Make clear which confounders were adjusted for and why they were included | | 18 | | When adjusted for age, sex, and vascular risk factors, multivariable logistic regression identified male (OR, 1.99; 95% CI, 1.30 to 3.05; P=0.002), history of CHD (OR, 1.85; 95% CI, 1.03 to 3.32; P=0.041), multiple acute infarcts (OR, 4.18; 95% CI, 2.07 to 8.45; P<0.0001), and territorial infarct (OR, 2.23; 95% CI, 1.52 to 3.27; P<0.0001) was more often associated with ICA territory disease. All parameters that were significant by univariate analysis at *P*<0.05 level or likely to have pathophysiologic influence were included in the multivariable logistic regression analysis. |
|  |  |  |  |  | (*b*) Report category boundaries when continuous variables were categorized | | 13, 15, 17, 19 | | Age ≥65 years; Prestroke mRS ≧2; Discharge mRS ≧2; Admission NIHSS >3; Discharge NIHSS >3. |
|  |  |  |  |  | (*c*) If relevant, consider translating estimates of relative risk into absolute risk for a meaningful time period | | N/A | |  |
| Other analyses | 17 | | Report other analyses done—eg analyses of subgroups and interactions, and sensitivity analyses | | | | N/A |  | |
| Discussion | | | | | | | | | |
| Key results | 18 | | Summarise key results with reference to study objectives | | | | 20 | In this study, we found that ICA disease group has more serious clinical and radiologic manifestation, and poorer outcome compared to MCA disease group. | |
| Limitations | 19 | | Discuss limitations of the study, taking into account sources of potential bias or imprecision. Discuss both direction and magnitude of any potential bias | | | | 23 | There are some limitations in our study | |
| Interpretation | 20 | | Give a cautious overall interpretation of results considering objectives, limitations, of analyses, results from similar studies, and other relevant evidence | | | | 20，21 | Our study found that IS in MCA territory is far more common than that in ICA territory. Previous study found that; | |
| Generalisability | 21 | | Discuss the generalisability (external validity) of the study results | | | | 23 | Research results from the large, prospective, multicenter, cohort study are helpful to understand the differences between ICA and MCA disease, and to improve the diagnosis and treatment of these patients. | |
| Other information | | |  | | | | | | |
| Funding | 22 | | Give the source of funding and the role of the funders for the present study and, if applicable, for the original study on which the present article is based | | | | 24 | This work was supported by the Project of | |

*Give information separately for cases and controls in case-control studies and, if applicable, for exposed and unexposed groups in cohort and cross-sectional studies.

**Note:** An Explanation and Elaboration article discusses each checklist item and gives methodological background and published examples of transparent reporting. The STROBE checklist is best used in conjunction with this article (freely available on the Web sites of PLoS Medicine at http://www.plosmedicine.org/, Annals of Internal Medicine at http://www.annals.org/, and Epidemiology at http://www.epidem.com/). Information on the STROBE Initiative is available at www.strobe-statement.org.
